# Acknowledgement to Reviewers of *Microarrays* in 2014

**DOI:** 10.3390/microarrays4010001

**Published:** 2015-01-09

**Authors:** Microarrays Editorial Office

**Affiliations:** MDPI AG, Klybeckstrasse 64, CH-4057 Basel, Switzerland

The editors of *Microarrays* would like to express their sincere gratitude to the following reviewers for assessing manuscripts in 2014:

Alliey, Ney
Brock, Guy
Calza, Stefano
Carpentieri, David
Castiglioni, Bianca
Cattoretti, Giorgio
Cavallini, Erika
Chechik, Gal
Chen, Guoan
Chen, Jin
Chen, Wenjin
Chevolot, Yann
Cretich, Marina
Dellinger, Andrew
Dembélé, Doulaye
Domany, Eytan
Dübel, Stefan
Dussert, Stéphane
Fededa, Juan Pablo
Gagné, François
Galbiati, Silvia
Gandhiraman, Ramprasad
Ghosh, Gargi
Gillett, Cheryl
Griffin, Robert J.
Jeffries, Marlo K. Sellin
Jungbluth, A. A.
Kadara, Humam
Kalari, Krishna
Kato, Takamitsu A.
Kaushik, Ajeet
Kelmansky, Diana
Labarge, Mark
Laurent, Louise C.
Lee, K. F.
Lei, Thomas K. F.
Lu, Lingeng
Malik, Karim
Manzano, Marisa
McCall, Matthew
Melamed, Jonathan
Päkkilä, Henna
Park, Chail-Il
Pasquer, Frédérique
Perkins, Edward
Pichler, Martin
Pirona, Raul
Prieto, Carlos
Rao, Uma N. M.
Ricardo, Sara
Scherer, Andreas
Sheridan, Paul
Sobek, Jens
Sugimura, Haruhiko
Townley, Helen E.
Usui, Kenji
Vaudry, David
Wineinger, Nathan
Wright, Gavin J.
Wu, Wei-Zhong
Zanella, Fabian
Zhang, Tongwu
Zhu, Xiangdong
Zilliox, Michael J.
Zlobec, Inti
Zuccheri, Giampaolo

